# Indication of Disrupted Temporal Structure in the Case of Thought Blocks in Schizophrenia: The Role of the Metastable Balance

**DOI:** 10.1155/2018/4031207

**Published:** 2018-09-02

**Authors:** Elias Koutsoukos, Elias Angelopoulos

**Affiliations:** ^1^Medical School, 1st Psychiatric Department, Eginition Hospital, Athens University, 72-74 Vas. Sofias Ave., 115 28 Athens, Greece; ^2^Signal Processing Laboratory, University Mental Health Research Institute, 2 Soranou Efessiou, 115 27 Athens, Greece

## Abstract

This study is aimed at investigating probable disruption of the metastable balance relevant to a disruption of the mental processes observed in the neurophenomenal level. This disruption was found to occur under dense auditory verbal hallucinations (AVHs) which are accompanied by thought blocking (TB) phenomena. The entropy that quantifies the complexity of the spontaneous coupling has been used to describe the observed transitions. According to our findings, the high synchrony-derived entropy (SE) defines a metastable state, where formations of cortical areas are able to coordinate transiently under the demands of stimulus-oriented processes or other internal cognitive associations. It was also found that the disruption of the sensitive balance to the side of oversynergy (overconnectedness) rather than the side of independence (coincidental coupling) is relevant with functional fixations under the specific symptom of schizophrenia. An introduced measure relative to the persistence of coupling indicated that the overcoupled brain areas exhibit a kind of “stiffness” in processing incoherent phasic components. Our consideration enhances the understanding of the role the metastability plays in the interpretation of deeply subjective phenomena, such as AVHs and TBs that affect the normal information routing in the brain.

## 1. Introduction

The oscillatory activity of the brain reflects the collective action or the functional convergence of neuronal subunits that are interacted in a larger set. The coherent activity is considered to serve either the local or the large-scale neuronal communication. The strength and duration of this neuronal coordination is modulated by various cognitive demands. The origin of these demands can be either stimulus-specific conveying information about their cause or internally induced from processes that reflect contents of perceptions, thoughts, imagination, and various mental states. This coordinative mechanism may allow different perceptual features of an object, different aspects of a moving scene, separate remembered parts of a significant experience, and even different ideas that arise in a conversation to be bound together into a more associative-defined entity [1–3]. Coordination dynamics imply the availability and capacity of the brain cites to collectively act under the demands of stimulus-oriented processes or other internal cognitive associations. Under this consideration, metastability is defined as a state where individual parts of the brain preserve their tendencies to function autonomously and at the same time to exhibit coordinated activity [4–8]. In this state, coherent and incoherent neuronal activities occur in a critical region between segregation and integration, respectively, a condition that exhibits similarities with the thermodynamic phase transitions observed in an excited system [3, 9]. In general, if these transitions (metastable state) are involved in the continuous flow of mental processes and contribute in the formation of new information, they should be detected under conscious states, reflecting a dynamical view of the coupling behaviour. Furthermore, irregularities of these transitions may interpret the abnormal brain functioning observed in nosological entities such as schizophrenia [10–17].

Considering the above, the present study is aimed at investigating probable disruption of metastability associated with the disruption of the mental processes occurred during the rare symptom of thought blocking in schizophrenia or in other words the consistency of phenomenal and electrophysiological findings. Thought blocking (TB) is a disorder of thought, usually induced by underlying psychotic processes. The phenomenon is characterized by regular interruptions in the stream of thought. Bleuler [18] described that “the phenomenon is developed rapidly and occurred in transitory episodes of varying duration, with blocking not only the stream of thought but also the entire psyche, involving the processes of attention, perception, memory, speech, and motility.” In the psychiatric bibliography, TB symptoms constitute core symptoms of schizophrenia and are attributed to the form and not to the content of the thought process [19]. These symptoms are rare and should be differentiated from the spontaneous distraction in the flow of thought occurring in normal individuals [20]. In our experience [21], phase synchrony analyses during dense and persisting auditory verbal hallucination (AVH) states that led to TBs have shown prolonged periods of strong coupling among the left temporal area and the remaining central and frontal cortical areas. The observed common mode of synchrony fluctuation lasted from 0.5 to 2 seconds, and it had been detected in a time window of 4 seconds referring to the denotation of the subjective experience of the symptom. The observation was found to engage, mostly, at the left temporal area and the remaining central and frontal brain areas. In this work [21], the methodological procedure was focused on the immediate vicinity of the symptom (symptom capture approach) and not on long-lasting resting-state EEG samples, which is the case of the present analysis.

In the present study, we examined the profile of the resting-state spontaneous coupling of EEG samples obtained from (a) normal healthy subjects, (b) a set of surrogates (see Methods section), (c) subjects suffering from persisted auditory verbal hallucinations and TBs (TB patients), and (d) a set of non-TB patients. The synchrony-derived entropy has been applied to quantify the complexity that governs the distribution of the average phase coherence (APC) values obtained from long-length resting-state EEG. As a rule, the APC computation, for all the included in the study samples, has been performed in segments of variable length, by giving us the ability to investigate the dependence between the APC values and the length of a given time window. The introduced approach allowed us to evaluate the persistence of coupling by means of the “rate of coupling loss,” a parameter relevant to the temporal characteristics of the phase locking. From our findings, there are indications that AVH and TB symptoms are disturbances in the connectivity level that concern unregulated excessive coordination. This functional overintegration was found to be relevant to functional fixations that led to the inhibition of processing noticed in the neurophenomenological level. The observation is similar with a disruption in the temporal structure to the side of overconnectedness. In the same line of evidence, disruption of the coordination mechanism, to the side of independence, may contribute in the disorganization syndrome in schizophrenia [4, 17, 22]. Both disturbances may be considered as irregularities of a fragile equilibrium between the segregated and collective functioning that envelope the metastable regime.

## 2. Methods

The TB patient group consisted of data based on recordings from twelve patients (5 males, 7 females, mean age: 32 ± 5, duration of illness: 13 ± 4 years, mean PANSS score: 78 ± 3), with drug-resistant spontaneous AVHs and with prominent experience of TBs during the recording periods. The non-TB patient group included recordings from twelve schizophrenic patients (8 men, 4 women, mean age: 33 ± 7; duration of illness: 12 ± 7 years; mean PANSS score: 65 ± 7) who, at the time of enrollment, did not exhibit AVH and TB symptoms. All subjects were seated in a light and sound-attenuated, double skin Faraday cage. Electrodes (Fp1, Fp2, F7, F3, Fz, F4, F8, FT7, FC3, FCz, FC4, FT8, T7, C3, Cz, C4, T8, TP7, CP3, CPz, CP4, TP8, P7, P3, Pz, P4, P8, O1, Oz, O2, and earlobes as mean electrical reference) were configured on the scalp, using a standard cap. Recordings of the horizontal-plane eye-movement potentials were made by two electrodes fixed 1 cm bilateral to the outer canthus of each eye. The skin resistance of each electrode was kept below 10 kΩ for the entire recording session, and the sampling rate was set to 500 samples per second. The recruitment of patients, the criteria for their inclusion in the present study, and the EEG data pretreatment have been previously described in detail [10, 21]. Additionally, EEG samples from twelve healthy volunteers (7 males and 5 females with mean age of 30 ± 5 years) have been included in the group of normals (labelled normals). As a rule, three recordings with 4 min duration for each corresponding participated group case have been segmented in a considerable number of epochs, covering the entire resting-state recording. A series of test intervals ranging from 400 to 2000 ms have also been considered in our analysis, while the standard segmentation width for the computation of APC was kept at 1000 ms for the entire study. Due to the robustness against the influence of noise, APC (see (3)) could be considered as a stable measure for phase synchronization in biological time series such as the EEG [23]. The following (1) and (2) are the analytic representations of two time series *x*(*t*) and *y*(*t*) following the Hilbert transformation:

(1) $$\begin{array}{c} z_{x}\left(t\right)=x\left(t\right)+j\tilde{x}\left(t\right)=a_{x}\left(t\right)e^{j\phi_{x}\left(t\right)}, \end{array}$$

(2) $$\begin{array}{c} z_{y}\left(t\right)=y\left(t\right)+j\tilde{y}\left(t\right)=a_{y}\left(t\right)e^{j\phi_{y}\left(t\right)}, \end{array}$$

where $j\tilde{x}\left(t\right)$ and $j\tilde{y}\left(t\right)$ are the imaginary parts of the complex variables *z*_*x*_(*t*) and *z*_*y*_(*t*), respectively, and *a*_*x*_ and *a*_*y*_ and *ϕ*_*x*_(*t*) and *ϕ*_*y*_(*t*) are the instantaneous amplitude and phase, respectively [24]. The measure *R* (see (3)) expresses the average phase coherence of a couple of *N* point segments, with 1 and 0 to be the limits of a strong and a weak coupling, respectively [24].

(3) $$\begin{array}{c} R=\left|\frac{1}{N}\overset{N-1}{\underset{k=1}{\sum }}e^{j\left(\phi_{x}\left(n\right)-\phi_{y}\left(n\right)\right)}\right|. \end{array}$$

An equal number of surrogate data sets have been also analyzed in the same manner as the original EEG data. The applied method was based on the rank-shuffled surrogate, a procedure that affects the temporal structure by generating artificial data sets by randomly shuffling the rank of the original signals [25]. We have also examined the efficacy of the followed procedure in the evaluation of phase synchrony data following the study of Sun et al. [26]. Finally, the distributions of the APC values, obtained from the different groups, have been subjected to entropy computations, introducing this way, a descriptor of the APC complexity, the synchrony-derived entropy (SE) based on Shannon's entropy [27].

(4) $$\begin{array}{c} SE=\frac{\sum_{k=1}^{n}s_{k}\log s_{k}}{\log\left(n\right)}, \end{array}$$

where *n* is the number of synchrony bins and *s* the synchrony values counted in each bin. SE constitutes a measure of information relative to the mode of coupling and could detect the preference of the connectivity for a given period. The high entropy values reflect flatness of the APC distribution denoting irregularity, instability, and lack of preference in the mode of coupling, while the low values are associated with prolonged phase locking.

The SE values that concern the TB patient group data were calculated from the resting-state EEG segments during prolonged periods (even several minutes) where dense hallucinatory states and TBs dominate. Always, the patients were instructed, immediately after the recovery from the thought blocking state, to annotate the past experience using an optical switch. This symptom capture approach was found more efficient in capturing isolated or sparse symptoms by considering the SE values found in association with the annotation [21]. After a detailed description of the experimental protocol, all subjects gave written informed consent and the University Mental Health Research Institute ethics committee approval was obtained. The experimental procedure was conducted in accordance with the Declaration of Helsinki [28].

## 3. Results

**Figure 1:**
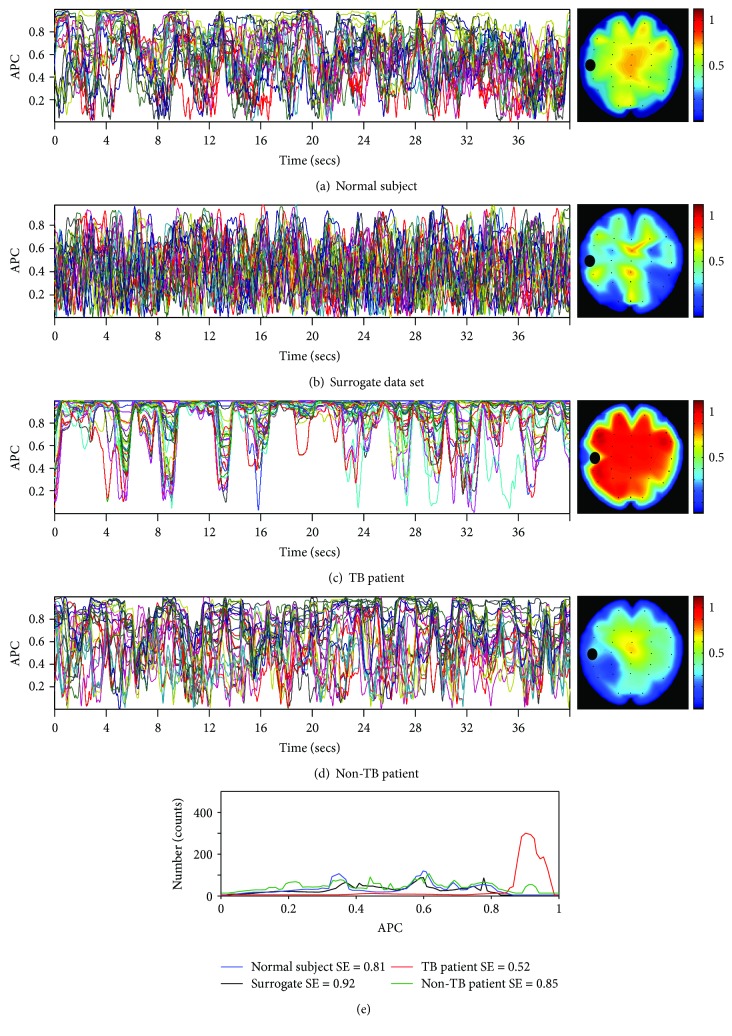
Representative resting-state APC values corresponding to a normal subject (a), a surrogate data set (b), a non-TB patient (c), and to a TB patient under persistent AVHs that led to TBs (d), respectively. Each line of plots represents the coupling between the left temporal region and the remaining brain sites during a representative 40 sec window. The spatial distribution of the coupling mode is shown for each case with an attached map to the right. The distributions of the APC values, measured for each case, are shown in (e). Synchrony-derived entropy values estimated for each of the four distributions are given under the graph, respectively.

In the present study, we investigate the continuous flow of patterns that the interconnected brain areas generate reflecting the instantaneous mode of connectivity. Four representative cases were chosen: a normal subject, a surrogate data set, a patient under dense AVHs who additionally exhibited TBs, and a non-TB patient, respectively.  Figure 1(a) represents the spontaneous variability of APC values corresponded to a 40 sec EEG segment obtained from a representative healthy subject. The phase coupling has been computed within the alpha EEG spectral region, among the left temporal, and the remaining brain sites. The attached map to the right indicates the spatial distribution of the observed coupling schemes, and it is compatible with the spatial characteristics of resting-state coupling under closed eye condition. The observation is congruent with the transient and nonstationary processes that characterize the normal brain functioning, and it was found to be common in all analyzed normal data sets. Accordingly, the estimated entropy (blue line appeared in Figure 1(e)) indicated a relatively flat distribution (SE = 0.81), denoting that the examined system does not exhibit any specific preference in the mode of coupling.

**Figure 2:**
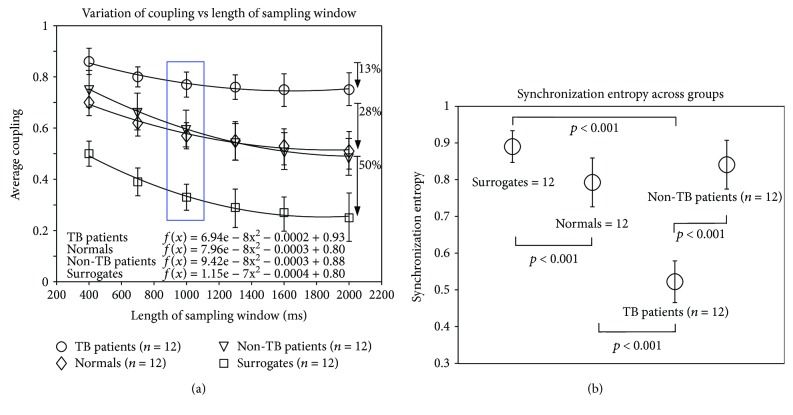
Average APC ± SD values versus length of the analysis window for TB patients, non-TB patients, normals, and surrogate group, respectively. (a) The fit models represent the rate of loss as a function of the length of the analysis window. The downward arrows indicate the “loss of coupling” as percentage of the initial values for each group, respectively. The blue frame includes the standard interval (1000 ms) values, where the differences among the four groups were found statistically significant. In (b), the average synchronization entropy values (SE ± SD) across the four groups are shown. Pairwise comparison of entropy values using the Mann–Whitney rank-sum test shows significant differences (*p* < 0.001) in the group pairs: normals-TB patients, normals-surrogates, surrogates-TB patients, and TB patients-non-TB patients.

Similarly, the spontaneous coupling obtained from a representative surrogate data set shows a morphological complexity analogous to a system, that is, its active units exhibit coincidental coupling (Figure 2(b)). The corresponding map indicated the lack of any spatial preference in the distribution of the coupling values. On the other hand, the distribution of synchrony values indicated a relative flatness (black line appeared in Figure 1(e)); analogously, the entropy value was found even higher (SE = 0.92) than in the normal subject.

The complexity of coupling is seen different from the other analyzed cases in the instance of the excessive and broadly distributed oscillatory states recorded from a representative patient under a dense hallucinatory state that led to thought blocking.  Figure 1(c) illustrates that several brain sites vary their resting-state coupling in a common mode with explicit preference to the high integration. As it is shown, the strong coupling was found lasted several seconds, while release of locking appeared intermittently. The distribution of APC values exhibited a peak relative to the prolonged temporal and spatial locking (red line appeared in Figure 1(e)). Accordingly, the estimated entropy during the majority of the persistent coupling instances was found within the range of 0.50 to 0.65 indicated, thus, a system having tendency to a relative stability. The attached map shows the spatial preference of the high coupling occurrences. It has to be noticed that in the majority of patient data, the coupling variability exhibited morphological similarities with the previously described representative case. The APC values appeared in Figure 1(d) concern the spontaneous variability of a non-TB patient. The formation did not show instances of persisting overcoupling, while the estimated entropy (green line appeared in Figure 1(e)) indicated a relatively flat distribution (SE = 0.85), denoting that the examined system does not exhibit any specific preference in the mode of coupling.

The dependence between the length of the analysis window (400 to 2000 ms) and the measured coupling, revealed different coupling behaviour among the four groups studied. The differences of the APC values for the standard interval (1000 ms) denoted by the blue box was found to be statistically significant (ANOVA *F*(3, 44) = 84.11, *p* < 0.001) (Figure 2(a)). Specifically, the difference between the non-TB patients and TB patients was found to be statistically significant (ANOVA *F*(1, 22) = 34.4, *p* < 0.001). The fitted lines define the “rate of synchrony loss” a parameter relevant to the amount of phasic components that could influence the balance between coherence-incoherence as a function of the length of the analysis window. The fast decay of the fitted lines indicates fast release of the coupling and vice versa. The group of surrogates exhibits the lower persistence since phase coherence is clearly symptomatic. The mean values of normals maintained a clear distance from the surrogates with the rate of loss found relatively lower. The non-TB patients indicated a fitted line relatively identical to that of normals. The TB patient data exhibited increased mean values of coupling well separated from the other groups, while the values observed in the long lengths indicated saturation of synchrony loss which is equivalent to prolonged coupling. More specifically, the difference between the non-TB patients and TB patients was found to be statistically significant (ANOVA *F*(1, 22) = 34.4, *p* < 0.001). This finding allowed us to associate the abnormal coupling (fixation) with the disruption of the mental flow, observed explicitly in the group of TB patients.

Additionally, pairwise comparison of synchronization entropy values across the four groups using the Mann–Whitney rank-sum test showed significant differences (*p* < 0.001) observed between arranged group pairs: normal-TB patients, normal-surrogates, surrogates-TB patients, and TB patients-non-TB patients, respectively (Figure 2(b)).

## 4. Discussion

Various complex systems, both natural and synthetic, exhibiting coherent and incoherent phenomena, have been computationally modeled using arrangements of weakly coupled oscillators [29, 30]. Simulation studies revealed a number of specific coupling behaviours and critical states that could influence the analysis strategies and consequently the understanding of brain functioning. The metastable chimera state based on the activity of community-organized oscillators has shown to generate a large repertoire of transient (metastable) states, where collective action and segregation coexist [31]. The abovementioned findings, although are in need of further theoretical understanding, exhibit similarities with those observed in the neurophenomenal level. In the brain level, functional segregation and integration are considered to be organizational aspects that reflect the collective and the independent activity of the neuronal components, respectively. These aspects also define the broader regions of irregularity and regularity, respectively [1, 4, 5, 7]. The normal brain functioning implies that the participating units retain their intrinsic mode of functioning (individuality and specificity), while at the same time these units participate collectively in transient schemes under various cognitive demands [17]. In this sense, a less defined and, thus, a more adaptive neuronal functioning could enhance the creation of new information. On the other hand, prolonged periods of excessive coherence (excessive synergy) in complex dynamical systems could be characterized as pathological. In the ground of a completely different neurophysiological-neuroanatomical mechanism, the severe oscillatory states occurred in the case of epileptic seizures were found to be associated with periods of impaired consciousness [13, 32].

The current work tries to reveal the involvement of the temporal organization, during a distinguished symptom of schizophrenia that led to interruptions of the thought. More precisely, we attempted to interpret the intermitted brain functioning in the phenomenal level, through specific irregularities of the metastable balance observed in the resting-state activity. TB symptoms are considered to be abnormalities of the flow of thought, and they could be conceived possibly as interruptions in the continuous information processing, due to the repetitive intrusions of hallucinations experienced by the subjects [21]. This point of view is in agreement to the theoretical consideration of Chapman [33] who stated that “the thought blocks are relevant with overload condition in the information processing.” The fact that the oscillatory mechanisms are affected during these symptoms of schizophrenia [10] led us to investigate the mode that excessive collective action is reflected the resting-state spontaneous coupling.

Several measures have been proposed to characterize quantitatively the interplay between the complementary tendencies of independent and collective actions. Tononi et al. [34] defined a statistical measure for the amount of “structure” within a system's dynamics, as expression of its “complexity” with its value being high for systems with subsets of large mutual statistical dependencies. In a later study by Tononi and Edelman [35], in the theoretical level, different parts of such systems can engage in separate activity and yet remain interdependent. An analysis, by Seth et al. [36], concerning the strengths and limitations of three quantitative measures of dynamical complexity in the neural systems underlying consciousness, neural complexity, information integration, and causal density, has shown that none of these measures alone could capture the multidimensional complexity of a working neuronal system. However, any approach that quantifies complex organizational aspects, such as segregation and integration, with a single descriptor, could be considered simplistic, and criticism is needed for the interpretation of the findings. As discussed by Kelso and Tognoli [1], the empirical characterization of a system's behaviour, in terms of transitory states, encounters some difficulties. It is crucial, before any attempt to find signatures of a metastable state, to identify from the continuous stream of brain activity (flow of mental processes) some segments corresponding to specific neurophenomenological regimes. Thus, in our study, the phenomenal disruption has been associated with a pathological connectedness. The synchronization entropy findings provide a statistical description of brain connectivity by measuring the average amount of information in a probability distribution. In our study, this descriptor was found to be sensitive enough in detecting changes in the temporal structure of brain functioning. The continuous fluctuation of coupling values, as denoted by the high SE values, reflects the widespread oscillations across the distributed cortical areas and suggests the nonstationary nature of the brain. This could be an interim state where both segregation and integration coexist by serving the availability of the neuronal assemblies to participate in the production of mental information. In the case of thought blocks (TB patients), the excessive production of information as defined by the notably low entropy values was found to restrain significantly the normal flow of mental contents. Although a common nosological background exists, the non-TB patients did not exhibit active symptoms (AVHs, TBs), neither the previously described erroneous mode of coupling. Referring to the disrupted connectivity, the disorder of the metastable sensitive balance between large-scale integration and independent processing in the cortex, in favor of independent processing, has been suggested to be involved in the disorganization syndrome in schizophrenia [11]. Schizophrenia is a multifaced disorder including delusional, hallucinatory, and formal thought disorders. These symptoms seem to be based on the background of disturbed connectivity which allows the manifestation of erroneous and autonomous symptoms that leads to phenomenal abnormalities in the stream of consciousness [37].

The domination of the frontotemporal excessive coupling (see Figure 1(c)), during the experience of TB symptoms, was found to restrain the spontaneous synchrony variability, impeding the continuous information processing. The prolonged phase locking implies that the coordinated cortical sites emerge and relay within frontotemporal areas uniformly unchanged informational contents. Map appeared in Figure 1(c) illustrates persistent coupling, possibly caused by failure of the feedback regulatory mechanism to manage the excessive coupling and in extend to the organization of the rhythmical activity. In this view, the understudied system became more stable, incapable to produce information, and consequently slothful. The low entropy findings indicated lower complexity. This assumption is in agreement with a shift from the metastable regime, characterized by transient patterns of synchrony, to episodes of prolonged stability, characterized by stationary coupling. In general, a synchronization index reflects the ratio between the coherent and incoherent contents of two activities assessed in a given time window. Based on the transitory nature of brain coupling, it is assumed that high spontaneous synchrony should be principally measured, in short time windows. Along this line of thinking, the introduced analysis regarding the persistence of coupling, allowed us to classify the different coupling preferences of the four groups. More specifically, the persistent cortical connectedness, under the specific symptoms of TB patients, indicated that the overcoupled brain areas exhibited a kind of “stiffness” in processing incoherent phasic components. This state is associated with the fixation of the functional specialization due to overintegration. The fact that the coupling profile of the non-TB patients was found to be clearly separated from that of TB patients supports further the existence of a common ground between the abnormal coupling and the findings at the phenomenal level. According to our findings, synchrony-derived entropy effectively describe the disruption occurred in a critical equilibrium between the segregated and collective functioning under abnormal coupling states. This consideration may contribute in the understanding of the role that metastability plays in the interpretation of deeply subjective phenomena related with the diminished processing of information in the brain level.

## Data Availability

The data used to support the findings of this study are available from the corresponding author upon request.
